# Does the Type of Electrotherapy Impact the Effectiveness of Complex Physiotherapy Administered to Individuals with Neck Pain?

**DOI:** 10.3390/jcm15103884

**Published:** 2026-05-18

**Authors:** Jolanta Zwolińska, Aleksandra Kielar, Marta Kasprzak

**Affiliations:** 1 University of Rzeszów, Faculty of Health Sciences and Psychology, Rejtana 16C, 35-959 Rzeszów, Poland; 2 St. Queen Jadwiga’s Regional Clinical Hospital in Rzeszow, ul. Lwowska 60, 35-301 Rzeszów, Poland; 3 University of Rzeszów, Faculty of Medicine, ul. Rejtana 16C, 35-959 Rzeszów, Poland

**Keywords:** neck pain, physiotherapy, electrotherapy, TENS, Träbert current

## Abstract

**Background**: The widespread prevalence of neck pain (NP) is a serious healthcare and social problem, and the question of which components of physiotherapy are most effective is still valid. **Objectives**: The objective of this study was to assess the effect of the type of electrotherapy applied on the outcomes of complex physiotherapy administered to individuals with NP. **Methods**: In line with the study protocol, 100 individuals with NP were enrolled and randomly divided into four groups. All groups received kinesiotherapy and phototherapy. Additionally, each group also received electrotherapy treatment, which was a differentiating factor. Participants were assessed at baseline, post-intervention, and after six months. The examination involved evaluation of pain using VAS and measurement of the cervical range of motion (ROM). Overall, seven parameters were assessed during each examination. **Results**: Pain intensity decreased in all individuals across the three study periods. A large effect size and changes exceeding the Minimal Clinically Important Difference (MCID) were observed only in the electrotherapy groups. The improvement in cervical spine ROM was comparable in the HF and LF TENS groups in the short- and long-term perspectives; however, a greater number of effects (*p* < 0.05) was observed in the HF TENS group. TC resulted only in large and moderate short-term effects reflected by improvements in cervical spine ROM. In the PLACEBO group, moderate long-term effects were observed. **Conclusions**: Low-frequency currents appear to improve the analgesic effectiveness of complex physiotherapy implemented in individuals with NP. TC may provide better short-term effects compared to long-term effects reflected by improvements in cervical spinal ROM. The effects in the PLACEBO group may suggest that phototherapy and kinesiotherapy are more effective due to the continuation of exercise and the education in ergonomics of work.

## 1. Introduction

As a result of technological advancement and digitalization, nowadays computers and smartphones are commonly used for work, study, and leisure [1,2]. It has been shown that there is a link between the use of electronic devices and the incidence of musculoskeletal symptoms, including cervical problems [1,3,4,5]. This relationship has also been confirmed in university students [2,6].

According to the 2015 Global Burden of Disease Study, spinal conditions are a significant health challenge, with neck and lower back pain identified as a leading cause of disability. Over the past decade, there has been an 18.7% increase in the prevalence of this type of pain [7]. At present, neck pain (NP) is one of the most common musculoskeletal conditions, with an incidence rate of 27.0 cases per 1000 people in 2019 [8]. According to other authors, neck pain is experienced by 10–24% of the general population, and the problem affects between 11% and 14% of all workers each year, leading to absences from work and poor quality of life [9].

Neck pain is defined as any pain located in the posterior part of the cervical spine that extends from the occipital condyles to the spinous process of the first thoracic vertebra. This pain may radiate to the shoulder and the area above the upper border of the clavicle and the suprasternal notch [10,11,12].

It has been shown that NP can effectively be reduced by various forms of conservative treatment, such as pharmacotherapy, health education, and regular physical activity, including exercise (stretching, strengthening, stabilization, and endurance training) and massage [13]. Electrotherapy and phototherapy are applied as adjunctive treatment methods in individuals with NP because, by using the physical agents, it is possible to reduce pain and facilitate motor improvement while reducing pharmacotherapy [14,15,16]. The pain-reducing effect of electrotherapy is explained by the gate control theory proposed by Melzack and Wall (Melzack & Wall, 1965) [17,18]. The analgesic effect also results from the release of endogenous opioid peptides [17,18,19,20,21,22].

The physical agents commonly applied in individuals with NP include transcutaneous electrical nerve stimulation (TENS), as well as TC, also referred to as Ultra-Reiz current or the 2–5 current [23,24,25]. There are two main TENS methods: HF TENS (∼50–100 Hz), with low intensity, and LF TENS (∼2–4 Hz), with high intensity, similar to acupuncture [26]. TENS affects subjective perception of pain, reduces muscle tension, and improves spinal mobility. Treatment with the use of TENS provides rapid effects that are sustained for several weeks or months [26,27,28]. TC provides a vibration-like effect that imitates massage and relaxes the tissues. The therapeutic effects of TC are associated with analgesic activity, reduction in muscle tension, improvement of circulation, and regulation of sympathetic activity of the autonomic nervous system [29,30]. A study has also shown that TC produces a significant increase in the parameters of the parasympathetic vagus nerve, and this seems to confirm the positive effects of TC on the human body [31].

Young people are also increasingly affected by NP, due to which there is a need for therapeutic programs that effectively reduce discomfort without compromising the individuals’ ability to perform daily activities and duties at work. These programs should include a variety of physiotherapy components as well as methodological guidelines useful to clinical practitioners.

A review of the literature also suggests that there is a lack of strong evidence showing the effectiveness of specific components of physiotherapy, for instance, in the combined use of electrotherapy and exercise in individuals with NP [32,33,34,35,36]. There is also a clear lack of research on the side effects of complex physiotherapy programs, including electrotherapy and therapeutic exercise. Furthermore, some studies do not present clearly defined parameters for the applied electrotherapy (pulse width, pulse shape, and pulse frequency) [37,38].

Moreover, there is a lack of direct comparisons between TENS and TC. Importantly, these methods operate based on different physiological mechanisms: TENS primarily targets the gate control theory of pain, whereas TC produces strong stimulatory effects impacting local circulation.

Given the limited number of studies investigating the clinical application of TC, clinicians and physiotherapists still do not have a definitive answer to which modality provides superior “added value” when combined with standard exercise programs.

This study aimed to assess the effect of a specific type of electrotherapy on the outcomes of complex physiotherapy applied to individuals with NP. In addition, the authors attempted to assess the potential risks associated with the use of complex physiotherapy in individuals with NP.

## 2. Materials and Methods

### 2.1. Ethics Approval

This study was conducted in compliance with the Declaration of Helsinki, and its protocol was approved by the Specialist Team for Ethics in Physiotherapy Research at the Polish Chamber of Physiotherapists (resolution No. 13/2022). Participants gave written informed consent before the data collection began. The study protocol was registered at clinicaltrial.gov as NCT04890743. The day of first registration was 29 April 2021.

### 2.2. Study Design

This study was conducted and reported according to the CONSORT guidelines. This was a randomized controlled trial with an exploratory character. Study participants meeting the inclusion criteria were randomly allocated to four groups in a double-blinded design. An identical basic physiotherapy program was applied as a standard for all groups. The differentiating factor was the type of electrotherapy administered in specific groups (the HF TENS group, the LF TENS group, the TC group, and the PLACEBO group).

### 2.3. Randomization and Blinding

Participants were randomly assigned to one of the four groups. The randomization process, enabled by computer-generated numbers, was carried out by members of the Student Science Club for Investigation of Physical Energy Used in Physiotherapy (SSC). Ultimately, 25 participants were allocated to each group. The study participants and the members of the SSC who performed the assessments of the participants were not informed about the type of electrotherapy applied in each case. The technique applied in this study to perform electrotherapy in the PLACEBO group was identical to that used in the other groups; however, the dose of current administered could not have affected the therapeutic outcomes. The room in which the treatments were administered was organized in such a way as to ensure that the study participants could not see the defined current parameters or the dose applied. The research team explained that reduced perception of electrical vibrations was observed in the PLACEBO group, compared to the other groups, owing to individual differences in sensitivity to electrical stimuli. The results of the randomization process were known to the supervisor of the SSC and to the person who had conducted the randomization procedure; however, they were not involved in the subsequent parts of the study. Members of the Science Club who administered the intervention in the specific groups were not engaged in the assessment of the study participants at any time point (Exams I, II, and III). The Science Club members who performed the procedures were unaware of the purpose and details of this study, nor did they know the results of the randomization. Furthermore, the persons conducting the measurements (Exams I, II, and III) were not involved in the therapeutic procedures. Their role was limited solely to the assessment of participants at specific time points. These individuals were also blinded to the results of the randomization.

### 2.4. Participants

This study was conducted in the Center for Innovative Research in Medical and Natural Sciences of the University of Rzeszów, and members of the SSC were therapists. The invitation to participate in the study was addressed to students of the University of Rzeszów who experienced NP. Written information was provided detailing the purpose and course of the study. Furthermore, the option available for the participants to withdraw from the study was emphasized at every stage. After being advised of the purpose and potential risks of the study, all participants provided written informed consent. A total of 102 individuals were willing to participate in the study. Ultimately, 100 individuals met the inclusion criteria and were randomly allocated to 4 groups: the HF TENS group, the LF TENS group, the TC group, and the PLACEBO group. The age of the participants was in the range of 20 to 26 years, with a mean age of 21.89 years.

### 2.5. Inclusion and Exclusion Criteria

The inclusion criteria were the following: (1) voluntary consent to participate in the study, (2) neck pain or limited cervical range of motion experienced for at least one month, (3) informed written consent of the individual to participate in the study, along with a statement about refraining from using painkillers or muscle relaxants during the observation period, (4) no contraindications for electrotherapy and phototherapy treatments administered in the cervical spine area, and (5) no history of cancer in the participant or their immediate family.

The exclusion criteria were as follows: (1) permanent anatomical changes in the cervical spine, (2) previous injuries to the spine, and (3) participation in intensive sports training.

### 2.6. Intervention

The procedures were performed from February 2024 to December 2024 in the afternoons and evenings by members of the SSC. Each stage of the study was monitored by a certified physiotherapist (SSC supervisor). A complex physiotherapy program was administered to all study participants; this included a specially designed kinesiotherapy program, phototherapy, and one of the three electrotherapy treatments or the PLACEBO treatment, depending on the outcome of the randomization process. The kinesiotherapy program included stretching exercises for the paraspinal muscles—especially the neck muscles—breathing exercises, correct posture training, and exercises to strengthen the paraspinal muscles and muscles of the shoulder girdle. The exercises were performed regularly, five times a week, each session taking approximately 30 min. During the first visit, each participant learned a set of twelve exercises. During the first meeting, the exercises were also modified for the specific participant; easier or more difficult variants were used depending on the participant’s functional status. The exercises were performed in two sets of 8–12 repetitions. The participants started with 8 repetitions and then, following consultation with the Science Club members, gradually increased the number so that by the third week they were performing 12 repetitions of each exercise.

Participants also received instruction related to ergonomics, with guidance on how to properly adjust the workplace to ensure optimum body posture and to minimize spinal loads. Participants also learned about relaxation techniques and exercises used at work to prevent cervical spine discomfort and improve daily functioning.

Over the course of three weeks, each participant completed 10 treatment sessions comprising a set of physical exercises, exposure to a Sollux lamp with a blue filter (intensity of irradiation to the point where they could clearly feel pleasant warmth), and, depending on group allocation, one of the three electrotherapies (up to the level of clearly felt and well-tolerated vibrations).

The LF TENS group received acupuncture-like LF TENS with a frequency from 2 Hz to 4 Hz and an impulse width from 50 μs to 150 μs (lengthwise placement of segmental electrodes with an area of 5 cm × 5 cm).

The HF TENS group received conventional HF TENS with a frequency from 60 Hz to 100 Hz and impulse width from 40 μs to 250 μs (electrode placement—see above).

The TC group received a treatment with Träbert’s current with a frequency of 142.8 Hz and impulse duration of 2 ms (electrode placement—longitudinal application: a cathode positioned cranially, at the level of C2–C3 for pain localized in the head area, and a cathode positioned caudally at the level of C6–C7 for pain radiating to the cervicothoracic junction).

The PLACEBO group received fake electrotherapy (acupuncture-like TENS with 0.1 mA current and electrode placement identical to both TENS groups).

Each treatment session lasted approximately one hour and included: phototherapy (20 min), kinesiotherapy (20 min), and electrotherapy (20 min).

### 2.7. Outcome Measures

Before the start of the treatment sessions, study participants completed a specially designed questionnaire that included personal data (age, gender, height, and weight). Participants performed a one-time self-assessment of their functional status using the Neck Disability Index (NDI) scores, which provided information on the intensity of neck pain and its impact on their performance in daily life (self-care, work, sleep, and recreation) [39].

The condition of the participants was assessed three times: before the start of the physiotherapy program (Exam I), at the end of the program (Exam II), and six months after the end of the intervention (Exam III). Besides that, each time before and after the therapy session, the participants were asked to report any adverse events (deterioration of mood, increased discomfort, or skin problems in the area where the treatments are applied).

The examination included:

- Subjective assessment of pain using a Visual Analog Scale (primary outcome);
- Measurement of cervical range of motion (secondary outcome).

The cervical range of motion was measured with the GULICK anthropometric tape in the sagittal (forward flexion and backward extension), frontal (right and left lateral bending), and transverse (right and left rotation) planes. Two independent raters measured the range of motion. The arithmetic mean was calculated from these two measurements.

### 2.8. Sample Size

To enroll participants for the study, the authors applied convenience sampling. For this purpose, the time and place of the study were specified, along with the organization of the academic year (winter and summer semesters) and the number of students at the University of Rzeszów meeting the adopted inclusion criteria. Taking all these aspects into account, the sample investigated in this study was of the largest size possible. Convenience sampling was applied since it was difficult to predict the exact number of individuals who would be able to fully participate in the study for such a long time. Due to the specific nature of the group (university students who also often work part-time), many potential participants were unable to guarantee their availability at all three assessment points. However, it was a priority for the research team to acquire long-term results, which are of key importance for clinical practice; because of this, it was necessary to accept a smaller but fully committed group constituting the largest possible sample that met the stringent temporal criteria. The requirement to attend the third examination six months after the procedure significantly reduced the number of individuals willing to participate in the project.

### 2.9. Data Analysis

A 0.05 significance level was adopted for the study. The analyses were computed using the R-4.4.0 software with the lme4 and lmerTest libraries. Since the investigated variables did not have normal distributions (according to the Shapiro–Wilk test), non-parametric methods were applied in the analyses. Comparisons of the values of quantitative variables across three or more repeated measurements were performed using the Friedman test. When significant differences between measurements were identified, the Wilcoxon matched-pairs tests with Bonferroni correction were performed as post hoc procedures to determine exactly which measurements differed. Comparisons of the values of the quantitative variable groups were performed using the Kruskal–Wallis test followed by the Dunn post hoc test. Patient ID and measurement time were considered random effects, and intervention was considered a fixed effect. The values of the qualitative variables in the groups were compared using the chi-square test (with Yates correction for 2 × 2 tables) or Fisher’s exact test when the assumptions of the chi-square test regarding the so-called expected counts were not met.

## 3. Results

### 3.1. Study Groups

**Figure 1:**
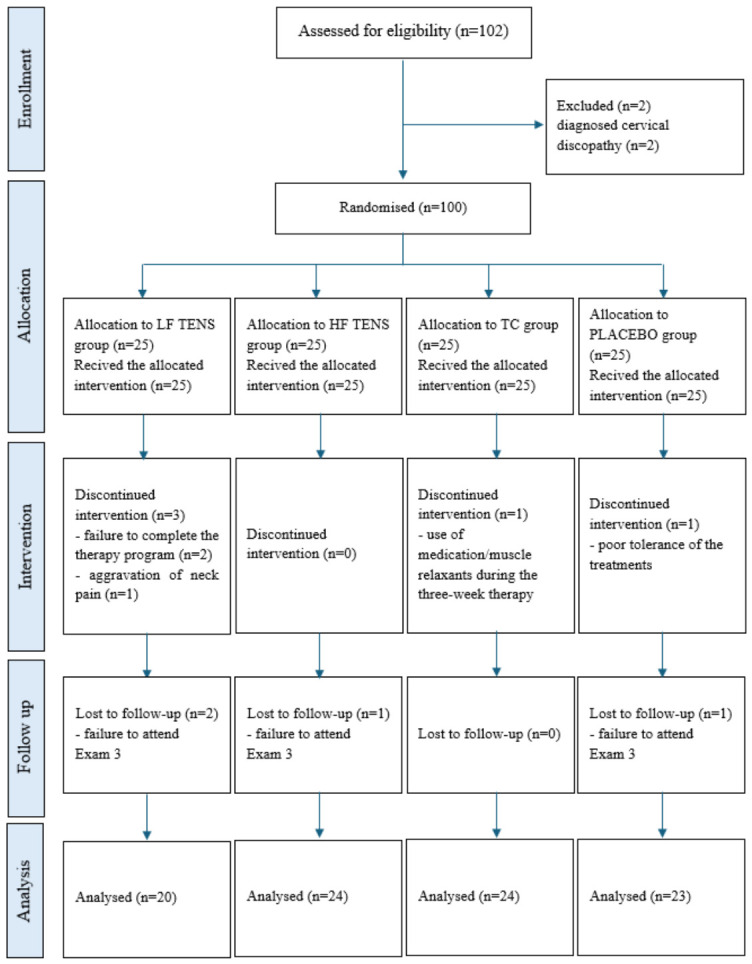
Detailed flow of study participants.

Nearly all students who reported NP and wished to participate in complex physiotherapy were enrolled in the study, with the exception of individuals with diagnosed discopathy. No contraindications to electrotherapy and phototherapy were identified in those who reported NP and wished to participate in the study. A gradual loss of participants was observed during the study. A total of nine individuals withdrew from the program. The reasons for discontinuing therapy included intolerance to the treatments administered, such as post-treatment skin changes (one person) and increased pain (one person). As a result, there was no contact with these participants during the subsequent stages of the study. One person began medication therapy during the intervention and withdrew from the study. During the intervention, two participants withdrew without providing a reason. Additionally, four individuals who had completed the intervention failed to attend the follow-up assessment, as a result of which they were excluded from the analysis. The detailed flow of the study participants is shown in Figure 1.

No differences were identified between the groups in relation to Body Mass Index (BMI). The NDI scores showed that the entire group of study participants included 12 individuals with moderate disability, 72 individuals with mild disability, and 7 individuals with no disability. There were no differences between the groups in the NDI (Table 1).

**Figure 2:**
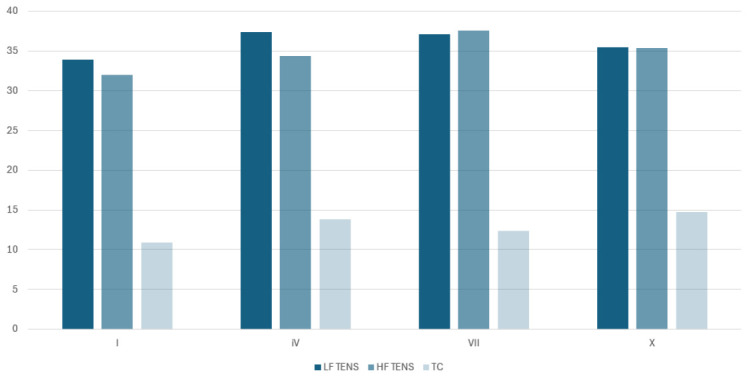
Mean current values in the study groups in the first, fourth, seventh, and tenth treatments.

Mild changes in current intensity were observed in the specific treatments in all groups (Figure 2).

**Figure 3:**
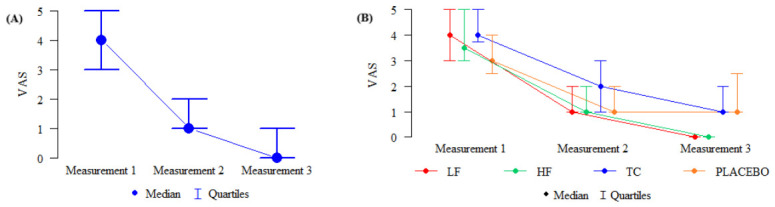
(**A**) Changes in pain intensity in all study participants. (**B**) Changes in pain intensity in the groups studied.

The following graphs present changes in pain intensity, measured using VAS at three consecutive points in time (Exam I, II, and III) (Figure 3).

### 3.2. Analysis of Changes in the Specific Groups

In the LF TENS group, after the intervention, a strong effect size (W = 0.925) was observed, reflected by pain reduction in short- and long-term periods (Table 2).

In the HF TENS group, after the intervention, a strong effect size (W = 0.936) was observed, reflected by pain reduction in short- and long-term periods (Table 3).

In the TC group, after the intervention, a strong effect size (W = 0.691) was observed, reflected by pain reduction in short- and long-term periods (Table 4).

In the PLACEBO group, only a moderate effect size (W = 0.422) was observed in the short- and long-term periods, reflected by a reduction in pain (Table 5).

In the LF TENS group, after the intervention, a moderate effect size, reflected by changes in the range of right rotation (W = 0.404) and left rotation (W = 0.369), was observed in the short- and long-term periods. Moreover, a moderate effect size reflected by the changes in right lateral flexion (W = 0.364) was observed in the short-term period (Table 2).

In the HF TENS group, after the intervention, a strong effect size, reflected by changes in flexion (W = 0.508) and right lateral flexion (W = 0.635), was observed in the short- and long-term periods. A moderate short- and long-term effect size was observed for left lateral flexion (W = 0.490). A weak short-term effect was observed for right rotation (W = 0.207), whereas a weak short- and long-term effect size was observed for left rotation (W = 0.294) (Table 3).

In the TC group, after the intervention, a strong short-term effect size was observed for right lateral flexion (W = 0.768) and right rotation (W = 0.629). A moderate short-term effect size was observed for flexion (W = 0.444), extension (W = 0.422), left lateral flexion (W = 0.334), and left rotation (W = 0.357) (Table 4).

In the PLACEBO group, after the intervention, a moderate long-term effect size was observed for right lateral flexion (W = 0.445) (Table 5).

### 3.3. Short-Term Effects of Therapy (Periods I–II)

In the short-term perspective, no statistically significant differences in pain reduction were observed between the study groups. However, the observed change in pain intensity exceeded the Minimal Clinically Important Difference (MCID) [40] threshold in all study groups except for the PLACEBO group. The MCID threshold was defined as a pain reduction greater than 1.7 points on the 10-point VAS scale (Table 6).

In the short-term perspective, improvement in mobility in the TC group was observed to be significantly greater compared to the other groups, with large effect sizes (η^2^ > 0.14) observed for five measured ranges of motion. In the TC group, improvement in left lateral flexion was also greater compared to the other groups; however, the effect size was moderate (η^2^ = 0.098) (Table 6).

### 3.4. Long-Term Effects of Therapy (Periods I–III)

In the long-term perspective, a significantly greater reduction in pain was observed in the LF TENS and HF TENS groups compared to the TC and PLACEBO groups, with an effect size of η^2^ = 0.277. Pain reduction also exceeded the MCID threshold in all study groups except for the PLACEBO group (Table 7).

In the long-term perspective, in the LF TENS group, a large effect (η^2^ > 0.14) and a significantly greater increase in two ranges of motion were observed: right lateral flexion and left lateral flexion, compared to the TC group. A large effect (η^2^ > 0.14) and a significantly greater increase in two ranges of motion were also observed for right rotation and left rotation compared to the TC and PLACEBO groups.

In the HF TENS group, compared to the TC group, a large effect (η^2^ > 0.14) and a significantly greater increase in two ranges of motion were observed: right lateral flexion and right rotation. Compared to the TC and PLACEBO groups, a significantly greater increase in flexion was also observed, with a moderate effect (0.06 < η^2^ < 0.14), as well as in left lateral flexion, with a large effect (η^2^ > 0.14). Additionally, a significantly greater increase in left rotation was observed compared to the PLACEBO group, with a large effect (η^2^ = 0.177).

### 3.5. Intervention Effects Compared with PLACEBO

Using a mixed-effects regression model, three intervention groups were compared with the PLACEBO group. The mean pain level (VAS) was 0.62 points higher in the TC group than in the PLACEBO group. Compared with the LF TENS group, the PLACEBO group demonstrated a lower flexion range of motion by 1.47 cm and greater left lateral flexion by 0.94 cm. Relative to the HF TENS group, the PLACEBO group showed lower flexion by 1.73 cm and lower extension by 0.86 cm, while left lateral flexion was greater by 0.65 cm. Compared with the TC group, the PLACEBO group demonstrated lower flexion by 1.53 cm, but greater right lateral flexion by 0.72 cm and greater left lateral flexion by 1.19 cm (Table 8).

## 4. Discussion

Neck pain is a complex biopsychosocial disorder affecting a significant part of the contemporary population. Projections suggest that NP will affect approximately 269 million people over the next 30 years, making it one of the major burdens on healthcare systems worldwide [41]. The widespread prevalence of cervical pain syndromes in contemporary populations creates a need to develop optimum therapeutic programs feasible in such cases [9]. Current research identifies exercise as the most effective physiotherapeutic factor in chronic neck pain [13,42,43].

Electrotherapeutic methods are easily accessible and safe interventions that reduce pain and disability by facilitating motor improvements in individuals with musculoskeletal problems [10,22,44,45,46,47,48]. To achieve significant therapeutic effects, treatments should be individually selected for each person [9,10,42]. As was mentioned earlier, some authors fail to provide detailed information about interventions. Some studies showed that electrotherapy applied in combination with exercise in patients with NP produced effects that were unrelated to the type of electrotherapy, and the use of the latter intervention did not provide significant gains compared to kinesiotherapy [34,49]. Clinical studies mainly focus on TENS treatments, and TC therapy has been investigated by some researchers as well [23,24,25,27].

In the present exploratory randomized study, the intervention included kinesiotherapy, phototherapy, and one type of electrotherapy (randomly assigned by the computer), the latter being a differentiating factor in the groups. LF TENS, HF TENS, and TC treatments were administered in compliance with the principles of electrotherapy methodology and safety, based on current guidelines for physiotherapists recommended for use in daily clinical practice [18]. In all participants, pain levels decreased systematically over the course of the study; however, the observed change exceeded the MCID only in the groups receiving electrotherapy. Additionally, an analysis of effect sizes indicated that strong effects were observed only in the groups receiving electrotherapy; in the group without electrotherapy, the effect was only moderate. According to Gedrimas and Aleknavičiūtė-Ablonskė [9], analgesic effects are produced by both high and low-frequency TENS (HF and LF TENS). The evidence confirming this statement was reported by Hernandez et al. (2021) [50], who observed reduced pain and disability in patients who received both LF TENS and HF TENS therapy in combination with dry needling.

Likewise, a study carried out by Martins-de-Sousa et al. [42,51] found no differences in the effects of LF TENS and HF TENS applied in combination with kinesiotherapy. According to the authors, the use of HF or LF TENS did not produce any additional clinical gains compared to PLACEBO treatment in patients with chronic neck pain. In contrast, the findings reported by Rodriguez-Huet et al. showed that the effectiveness of standard TENS is comparable to that of more advanced methods, such as Transcranial Direct Current Stimulation (tDCS), when it comes to pain reduction and improvement in the range of motion [52].

In the present study, improvement in cervical ROM appears to be comparable in the HF TENS and LF TENS groups. However, it should be emphasized that, in contrast to the LF TENS group, the HF TENS group demonstrated large effects reflected by improvements in some movements: flexion and right lateral flexion. The analysis of ROM changes also indicates a greater number of statistically significant effects (*p* < 0.05). TC provides only short-term effects expressed by improvements in cervical ROM; large and moderate effects with statistical significance (*p* < 0.05) were observed exclusively in the short-term period.

The current findings showed similar effectiveness of LF TENS and HF TENS, both short-term and long-term. However, HF TENS produced slightly better effects compared to LF TENS.

It is suggested that (conventional) HF TENS and (acupuncture-like) LF TENS should be applied with an intensity at a level of sensory threshold [9]. It has also been shown that greater intensity of TENS treatment produces better analgesic effect [20,35,53]. The literature review, however, shows that few studies have reported changes in the treatment intensity [10]. On the other hand, the recommended intensity of TC during treatments is 15–25 mA, and in some cases it may even reach 60–80 mA [30,44,54,55,56]. Some authors do not specify the intensity of TC applied or report an intensity at a level that is well tolerated by patients [25,30,45,46]. According to Poděbradský et al. [57], adaptation to TC does not take place even with prolonged use of the method, while den Adel et al. [58] pointed out that accommodation to TC occurs fairly quickly.

The optimum duration of a single electrotherapy session is a disputable matter [22]. It is usually 20 min [9], although some researchers apply a shorter (from 10 to 15 min) [44,45,56,59] or longer (30 min) duration [25,60,61]. In line with typical recommendations, 10–15 treatments should be administered over a course of two or three weeks [25,30,44,45,56,60]. Only in isolated studies did the authors apply one treatment per week [46], or three [62] to six [61] treatments in a series. In some cases, the authors themselves point out that the applied frequency of the treatments may be insufficient to produce a long-term analgesic effect [46]. In the current study, the intervention consisted of 10 therapeutic sessions conducted over a three-week period, with each electrotherapy session lasting 20 min. The findings show long-term pain reduction following the application of both LF TENS and HF TENS.

As suggested by the 44-year clinical experience of one of the co-authors of this research, it is important to adjust the treatment parameters to each person’s specific needs and sensitivity, and this approach was applied during the administration of electrotherapy in this study. The intensity of the treatments, i.e., the current intensity, was kept at the level of perceptible tingling and was reduced if the sensations were too strong or if there were any disturbing symptoms. In the three groups receiving electrotherapy, the current intensity was observed to be well tolerated by each participant. There was no regularity in the changes in current intensity in the successive treatments. However, the mean values in the tenth treatment were higher compared to the first treatment. Compared to the initial current intensity, an increase of 35.55% was observed in the TC group, as well as 4.7% and 10.65% in LF TENS and HF TENS, respectively. This observation may suggest that rapid accommodation to the vibrating sensation may be associated with the fact that the effects of a given electrotherapy are maintained for a short period of time. The other characteristics of the electrotherapy program applied, i.e., duration and number of treatments, were consistent with those most commonly suggested by other authors.

Other studies have shown that both TENS and interferential currents (IFC) have similar efficacy in the treatment of patients with NP. However, they do not provide additional gains for patients with NP if these therapies are used in combination with neck stabilization exercises (NSE) [49]. However, similar effectiveness of TENS and diadynamic currents (DD) has been shown in patients with lumbar disk disease and with low-back pain [63,64]. Additionally, Escartell Mayor et al. [33] reported that short-term gains from manual therapy applied in combination with exercise were similar to those produced by TENS applied in combination with the same type of exercise. The latter study applied isometric neck exercise [49], whereas in the current study, the therapy program included phototherapy, kinesiotherapy, and one of the three electrotherapy procedures. On the other hand, Dissanayaka et al. [65] reported that TENS therapy combined with standard physiotherapy is more effective in patients with myofascial pain syndrome (MPS) compared to IFC and PLACEBO. The effects obtained were immediate, yet they were only short-term.

In the current study, LF TENS, HF TENS, and TC therapy produced the desirable short-term effects. The changes observed between examination one and two were significantly better compared to the PLACEBO group. According to some authors, TENS therapy is less effective than IFC therapy [61,62]. On the other hand, Chwieśko-Minarowska et al. [30] reported poorer short-term effects of TC compared to manual massage. In contrast, the current study involved participants with NP, and in line with the study design, no manual therapy was applied. It is possible that this type of therapy would have been more effective; yet, in this case, the authors aimed to compare the efficacy of the selected electrotherapy methods in a research project that presented optimum safety and practical advantages.

In the present study, following a series of physical rehabilitation sessions, the PLACEBO group showed a moderate analgesic effect size (W = 0.442) in the short- and long-term periods and a strong effect size (W = 0.445) reflected by significant improvement in right lateral flexion in the long-term period. It can be assumed that these resulted from the use of phototherapy and kinesiotherapy. It is possible that the study participants followed the provided instructions and continued the exercises after the intervention had ended. This effect appears to partly confirm the findings reported by Sahshi & Sharma [66], showing that strengthening and mobility-enhancing exercises alone contribute to improved daily functioning in individuals with cervical spondylosis. A study by Suh et al. [67] showed that TENS therapy was more effective compared to the PLACEBO procedure [10]. Conducted later, the study by Martins-de-Sousa et al. [42] demonstrated that the use of both HF TENS and LF TENS as adjunct treatment in an exercise program does not enhance the therapeutic effect. A review of the literature suggests that the use of TENS therapy in individuals with NP is not more effective than exercise but appears to be more effective than PLACEBO [14,49]. Another literature review shows that the evidence supporting greater effectiveness of TENS in comparison to PLACEBO is insufficient [36].

The authors of the studies taken into account in the reviews report no adverse effects of electrotherapy in patients with musculoskeletal pain, including NP [22,36]. The review by Furlan et al. [68] shows that the most common undesirable effect of TC therapy was increased severity of pain reported by 1.5% to 25% of the study participants. In the present study, the participants were asked about any side effects experienced during the intervention. One participant reported an increase in discomfort, and another reported post-treatment skin changes at the electrode placement site. Consequently, these participants did not complete the therapy program and were excluded from further analyses. Findings reported by other authors show that both neck stabilization exercise (NSE) and NSE combined with electrotherapy make it possible to reduce pharmacotherapy in study participants [32,49,69]. The present study was conducted in a group of university students who were not using any medication. However, it is important to note that the demographic characteristics of our study population represent a specific cohort of young adults (mean age ~22 years) with predominantly mild functional disability. Consequently, the findings of this research may have limited external validity. While the results demonstrate the effectiveness of the intervention in this specific demographic, caution should be exercised when generalizing these conclusions to the broader population of patients with neck pain, particularly older individuals or those with significant comorbidities and advanced degenerative changes.

Focusing on this narrow population allowed for the assessment of electrotherapy’s impact in a model of non-specific neck pain, minimizing confounding factors like multi-morbidity or long-term structural spine pathologies. Nonetheless, future studies should include a more diverse patient population to confirm whether these specific electrotherapy protocols maintain their efficacy across different age groups and more severe clinical manifestations of neck pain.

One participant who used pharmacotherapy on a single occasion during the treatment period was also excluded from further analyses.

It seems that the attempt undertaken by the authors in this study to assess the benefits and potential risks of complex physiotherapy administered to individuals with NP was fully justified, and the objectives set at the start of the research project were achieved.

### 4.1. Study Limitations

According to some authors, it would be worthwhile to take into account psychosocial factors, such as depression, anxiety, quality of life, and work/employment, while evaluating the effects of therapy in individuals with NP [49,70]. The present study did not assess changes related to work since the participants were university students. Furthermore, during the interventions and assessments, the participants exhibited no symptoms of anxiety or deterioration of mood. The participants in the present study were young adults, mainly with mild disability, which, to some extent, limits the possibility of generalizing the findings; however, it should be emphasized that the currently available literature clearly shows that neck pain can affect people of all ages.

It should also be pointed out that the NDI questionnaire was used only once in order to assess the homogeneity of the groups prior to the intervention, due to which it was impossible to assess a potential change in the degree of disability after the intervention ended. However, the authors are planning to investigate the impact of the interventions on life satisfaction in the next phase of the research project, focusing on the use of various phototherapy methods in individuals with NP, and, in this case, the NDI questionnaire will be used at each stage of the study participants’ assessment.

Another limitation of this study is the absence of an intention-to-treat analysis. The study applied the per-protocol principle in the analyses.

### 4.2. The Strengths of the Current Study

As recommended by other authors, the intervention applied in this study comprised a uniform therapeutic exercise program in combination with two other treatments [22]. By doing so, the authors sought to adhere to the bioethical principle of beneficence by offering all study participants an intervention that would produce maximum gain for them [22,70].

It has been pointed out that many related studies present no comparative assessment of effects produced by TENS therapy applying different frequencies, and they do not assess long-term effects of the therapy [22,42]. Conversely, the current study has compared the effectiveness of low-frequency and high-frequency TENS and presents long-term effects assessed at a six-month follow-up. The assessment of long-term effects was a response to other authors’ suggestions that future studies should include the longest possible follow-up period [52].

The authors’ clinical experience suggests that generally the duration of the effects of complex physiotherapy programs administered to individuals with NP is varied and depends on the method and parameters of the treatments. Consequently, further well-designed research should investigate these factors. At the next stage of this research project, the authors intend to evaluate the effects of therapeutic exercise applied in combination with selected phototherapy methods. The authors believe such a comprehensive approach is needed, given the severity of the medical and social problems presented by neck pain syndromes.

## 5. Conclusions

Low-frequency currents appear to improve the analgesic effectiveness of complex physiotherapy implemented in individuals with NP. LF TENS and HF TENS seem to demonstrate satisfactory efficacy in the treatment of individuals with NP, which suggests that they potentially may be effectively used to reduce pain and improve cervical spine mobility. Träbert’s current may provide better short-term effects compared with long-term effects, as reflected in improvements in cervical mobility. Changes observed in several parameters in the PLACEBO group may indicate that phototherapy and kinesiotherapy are partially effective and that their effectiveness may also result from the continuation of exercise programs and the health education provided, including workplace ergonomics, relaxation techniques, and exercises performed at work.

Complex physiotherapy, including kinesiotherapy, phototherapy, and electrotherapy, implemented in individuals with neck pain seems to produce undesirable side effects only in isolated cases. This finding needs to be confirmed in future clinical trials with strictly defined parameters and treatment methodologies.

## Figures and Tables

**Table 1:** Study participants’ characteristics.

Parameter		LF (N = 20)	HF (N = 24)	TC (N = 24)	PLACEBO (N = 23)	Total (N = 91)	*p*
Age [years]	Mean (SD)	21.55 (1.88)	21.88 (1.26)	21.88 (1.33)	22.22 (1.17)	21.89 (1.41)	*p* = 0.212
	Median (quartiles)	21 (20–23)	22 (21–22.25)	22 (21–22.25)	22 (21–23)	22 (21–23)	
	Range	20–26	20–25	20–25	21–25	20–26	
BMI [kg/m^2^]	Mean (SD)	22.94 (3.25)	22.43 (2.54)	22.85 (2)	23.52 (2.98)	22.93 (2.69)	*p* = 0.677
	Median (quartiles)	21.61 (20.63–24.86)	21.96 (20.45–24.41)	22.15 (21.46–24.23)	23.03 (22.32–24.32)	22.53 (20.96–24.42)	
	Range	18.94–28.73	18.43–28.91	20.03–26.49	19.23–30.86	18.43–30.86	
BMI	Thinness	0 (0.00%)	0 (0.00%)	0 (0.00%)	0 (0.00%)	0 (0.00%)	*p* = 0.536
	Underweight	0 (0.00%)	1 (4.17%)	0 (0.00%)	0 (0.00%)	1 (1.10%)	
	Normal weight	15 (75.00%)	21 (87.50%)	21 (87.50%)	18 (78.26%)	75 (82.42%)	
	Overweight	5 (25.00%)	2 (8.33%)	3 (12.50%)	4 (17.39%)	14 (15.38%)	
	Obesity	0 (0.00%)	0 (0.00%)	0 (0.00%)	1 (4.35%)	1 (1.10%)	
	Obesity, class II	0 (0.00%)	0 (0.00%)	0 (0.00%)	0 (0.00%)	0 (0.00%)	
	Obesity, class III	0 (0.00%)	0 (0.00%)	0 (0.00%)	0 (0.00%)	0 (0.00%)	
NDI [pts]	Mean (SD)	10.85 (4.22)	10.17 (4.36)	9.58 (4.15)	7.65 (3.97)	9.53 (4.27)	*p* = 0.081
	Median (quartiles)	11 (9.75–13.25)	10 (7–12.25)	9 (7–13.25)	8 (6–9)	9 (7–13)	
	Range	2–18	2–20	1–19	0–16	0–20	
NDI-level	No disability	1 (5.00%)	1 (4.17%)	2 (8.33%)	3 (13.04%)	7 (7.69%)	*p* = 0.929
	Mild disability	16 (80.00%)	20 (83.33%)	18 (75.00%)	18 (78.26%)	72 (79.12%)	
	Moderate disability	3 (15.00%)	3 (12.50%)	4 (16.67%)	2 (8.70%)	12 (13.19%)	

*p*—Qualitative variables: chi-squared or Fisher’s exact test. Quantitative variables: Kruskal–Wallis test.

**Table 2:** Changes in the parameters in the LF TENS group in the successive measurements.

Parameter	Measurement	N	Mean	SD	Median	Min	Max	Q1	Q3	*p*
VAS	Measurement 1	20	4.10	1.02	4.00	3.0	6	3.0	5.0	*p* < 0.001 * W = 0.925 M1 > M2 > M3
	Measurement 2	20	1.40	0.68	1.00	1.0	3	1.0	2.0	
	Measurement 3	20	0.35	0.81	0.00	0.0	3	0.0	0.0	
Flexion [cm]	Measurement 1	20	3.95	0.71	4.00	3.0	5	3.5	4.5	*p* = 0.155 W = 0.093
	Measurement 2	20	4.50	1.05	4.00	3.0	7	4.0	5.0	
	Measurement 3	20	4.65	1.08	5.00	3.0	7	4.0	5.0	
Extension [cm]	Measurement 1	20	7.75	1.54	8.00	5.0	11	7.0	8.0	*p* = 0.22 W = 0.076
	Measurement 2	20	8.40	1.54	9.00	6.0	11	7.0	9.0	
	Measurement 3	20	7.80	1.36	7.00	6.0	10	7.0	9.0	
Right lateral flexion [cm]	Measurement 1	20	5.15	0.86	5.00	3.5	6	5.0	6.0	*p* = 0.001 * W = 0.364 M2 > M1
	Measurement 2	20	5.80	1.44	5.50	4.0	8	5.0	7.0	
	Measurement 3	20	6.10	1.97	5.50	3.0	9	5.0	8.0	
Left lateral flexion [cm]	Measurement 1	20	5.35	1.43	5.00	3.5	9	5.0	5.5	*p* = 0.411 W = 0.044
	Measurement 2	20	5.60	1.45	5.50	4.0	8	4.5	6.0	
	Measurement 3	20	6.10	1.62	5.50	4.0	9	5.0	8.0	
Right rotation [cm]	Measurement 1	20	7.85	1.17	7.25	7.0	10	7.0	8.0	*p* < 0.001 * W = 0.404 M3, M2 > M1
	Measurement 2	20	8.90	0.99	9.00	7.0	10	8.0	10.0	
	Measurement 3	20	9.00	1.03	9.00	7.0	10	8.0	10.0	
Left rotation [cm]	Measurement 1	20	7.30	1.72	7.00	4.0	10	6.0	9.0	*p* = 0.001 * W = 0.369 M3, M2 > M1
	Measurement 2	20	8.80	1.11	9.00	7.0	11	8.0	9.0	
	Measurement 3	20	9.20	1.20	10.00	7.0	10	9.0	10.0	

*p*—Friedman test + post hoc analysis (Wilcoxon paired tests with Bonferroni correction); * statistically significant (*p* < 0.05); W—Kendall’s W effect size; M1—Measurement 1, M2—Measurement 2, M3—Measurement 3.

**Table 3:** Changes in the parameters in the HF TENS group in the successive measurements.

Parameter	Measurement	N	Mean	SD	Median	Min	Max	Q1	Q3	*p*
VAS	Measurement 1	24	3.83	1.27	3.50	2.0	6	3.00	5.00	*p* < 0.001 * W = 0.936 M1 > M2 > M3
	Measurement 2	24	1.46	1.02	1.00	0.0	3	1.00	2.00	
	Measurement 3	24	0.08	0.28	0.00	0.0	1	0.00	0.00	
Flexion [cm]	Measurement 1	24	3.92	0.72	4.00	2.5	5	3.50	4.12	*p* < 0.001 * W = 0.508 M3 > M2 > M1
	Measurement 2	24	4.58	1.14	4.50	3.0	7	3.88	5.00	
	Measurement 3	24	5.38	1.27	5.00	3.5	8	5.00	5.50	
Extension [cm]	Measurement 1	24	8.08	0.86	8.00	7.0	10	7.50	8.25	*p* = 0.664 W = 0.017
	Measurement 2	24	8.42	0.97	9.00	7.0	10	7.75	9.00	
	Measurement 3	24	7.92	1.98	7.50	4.0	11	7.00	10.00	
Right lateral flexion [cm]	Measurement 1	24	4.92	0.69	5.00	4.0	6	4.38	5.12	*p* < 0.001 * W = 0.635 M2, M3 > M1
	Measurement 2	24	6.50	1.06	6.00	5.0	8	6.00	7.25	
	Measurement 3	24	6.38	1.49	5.75	5.0	9	5.00	8.00	
Left lateral flexion [cm]	Measurement 1	24	5.00	1.04	5.00	3.0	7	4.38	5.62	*p* < 0.001 * W = 0.49 M3, M2 > M1
	Measurement 2	24	6.33	1.20	6.00	5.0	8	5.00	7.25	
	Measurement 3	24	6.58	1.59	6.50	4.0	9	5.00	8.00	
Right rotation [cm]	Measurement 1	24	8.54	1.28	9.00	6.0	10	8.00	9.25	*p* = 0.007 * W = 0.207 M2 > M1
	Measurement 2	24	9.50	1.29	9.50	8.0	11	8.00	11.00	
	Measurement 3	24	9.08	1.21	9.00	7.0	11	8.75	10.00	
Left rotation [cm]	Measurement 1	24	8.50	1.41	8.50	6.0	11	7.75	9.25	*p* = 0.001 * W = 0.294 M3, M2 > M1
	Measurement 2	24	9.42	1.06	9.50	8.0	11	8.75	10.00	
	Measurement 3	24	9.50	0.98	10.00	7.0	11	9.00	10.00	

*p*—Friedman test + post-hoc analysis (Wilcoxon paired tests with Bonferroni correction); * statistically significant (*p* < 0.05); W—Kendall’s W effect size; M1—Measurement 1, M2—Measurement 2, M3—Measurement 3.

**Table 4:** Changes in the parameters in the TC group in the successive measurements.

Parameter	Measurement	N	Mean	SD	Median	Min	Max	Q1	Q3	*p*
VAS	Measurement 1	24	4.25	1.07	4.0	3	7	3.75	5.00	*p* < 0.001 * W = 0.691 M1 > M2, M3
	Measurement 2	24	2.38	1.24	2.0	1	5	1.00	3.00	
	Measurement 3	24	1.67	1.86	1.0	0	7	1.00	2.00	
Flexion [cm]	Measurement 1	24	3.88	0.95	3.5	3	5	3.00	5.00	*p* < 0.001 * W = 0.444 M2 > M3, M1
	Measurement 2	24	5.33	1.20	5.0	4	8	4.00	6.00	
	Measurement 3	24	4.08	1.82	4.0	2	10	3.00	4.25	
Extension [cm]	Measurement 1	24	6.54	2.21	6.0	4	11	5.00	8.25	*p* < 0.001 * W = 0.422 M2 > M3, M1
	Measurement 2	24	8.62	1.76	9.0	6	12	7.00	10.00	
	Measurement 3	24	6.71	2.24	6.0	3	10	5.50	9.00	
Right lateral flexion [cm]	Measurement 1	24	5.42	1.06	5.0	4	7	5.00	6.00	*p* < 0.001 * W = 0.768 M2 > M1 > M3
	Measurement 2	24	6.71	1.40	6.0	5	11	6.00	8.00	
	Measurement 3	24	4.33	0.70	4.0	3	6	4.00	5.00	
Left lateral flexion [cm]	Measurement 1	24	5.33	1.49	5.0	3	8	4.75	6.00	*p* < 0.001 * W = 0.334 M2 > M1 > M3
	Measurement 2	24	6.79	1.74	6.0	4	10	6.00	7.25	
	Measurement 3	24	4.17	1.37	4.0	3	7	3.00	5.00	
Right rotation [cm]	Measurement 1	24	8.46	1.67	8.0	6	12	8.00	8.50	*p* < 0.001 * W = 0.629 M2 > M1 > M3
	Measurement 2	24	9.62	1.53	9.5	6	13	9.00	10.00	
	Measurement 3	24	7.12	1.36	8.0	4	9	6.00	8.00	
Left rotation [cm]	Measurement 1	24	8.00	1.50	8.0	5	11	7.00	8.25	*p* < 0.001 * W = 0.357 M2 > M1, M3
	Measurement 2	24	9.96	1.65	10.0	8	14	9.00	10.25	
	Measurement 3	24	7.96	1.37	8.0	5	10	7.00	9.00	

*p*—Friedman test + post hoc analysis (Wilcoxon paired tests with Bonferroni correction); * statistically significant (*p* < 0.05); W—Kendall’s W effect size; M1—Measurement 1, M2—Measurement 2, M3—Measurement 3.

**Table 5:** Changes in the parameters in the PLACEBO group in successive measurements.

Parameter	Measurement	N	Mean	SD	Median	Min	Max	Q1	Q3	*p*
VAS	Measurement 1	23	3.22	1.04	3	1	5	2.5	4.0	*p* < 0.001 * W = 0.442 M1 > M3, M2
	Measurement 2	23	1.52	0.95	1	0	4	1.0	2.0	
	Measurement 3	23	1.70	1.40	1	0	5	1.0	2.5	
Flexion [cm]	Measurement 1	23	2.78	0.60	3	2	4	2.0	3.0	*p* = 0.104 W = 0.098
	Measurement 2	23	2.91	0.67	3	2	4	2.5	3.0	
	Measurement 3	23	3.00	0.67	3	2	4	3.0	3.0	
Extension [cm]	Measurement 1	23	7.48	0.99	7	5	9	7.0	8.0	*p* < 0.001 * W = 0.337 M2 > M3
	Measurement 2	23	7.43	1.16	7	4	9	7.0	8.0	
	Measurement 3	23	6.91	1.35	7	4	9	6.0	8.0	
Right lateral flexion [cm]	Measurement 1	23	5.70	1.18	6	4	8	5.0	6.0	*p* < 0.001 * W = 0.445 M3 > M2, M1
	Measurement 2	23	6.22	1.31	6	4	8	5.5	7.0	
	Measurement 3	23	6.70	1.26	6	4	9	6.0	7.0	
Left lateral flexion [cm]	Measurement 1	23	6.30	1.36	7	4	8	5.5	7.0	*p* = 0.247 W = 0.061
	Measurement 2	23	6.78	1.17	7	4	9	6.0	8.0	
	Measurement 3	23	6.78	1.24	7	4	9	6.0	7.0	
Right rotation [cm]	Measurement 1	23	8.70	1.69	8	6	12	8.0	10.0	*p* = 0.931 W = 0.003
	Measurement 2	23	8.57	1.88	8	6	12	7.5	10.5	
	Measurement 3	23	8.70	1.26	8	7	11	8.0	9.5	
Left rotation [cm]	Measurement 1	23	8.96	1.89	9	6	12	7.5	10.5	*p* = 0.105 W = 0.098
	Measurement 2	23	8.96	1.74	9	6	12	8.0	10.5	
	Measurement 3	23	8.70	1.26	8	7	11	8.0	9.5	

*p*—Friedman test + post-hoc analysis (Wilcoxon paired tests with Bonferroni correction); * statistically significant (*p* < 0.05); W—Kendall’s W effect size; M1—Measurement 1, M2—Measurement 2, M3—Measurement 3.

**Table 6:** Comparison of positive short-term effects in specific groups.

Parameter	Group	N	Mean	SD	Median	Min	Max	Q1	Q3	*p*
VAS decrease	LF	20	2.7	1.26	3	1	5	2	3.25	*p* = 0.062 η^2^ = 0.05
	HF	24	2.38	1.24	2	0	5	1.75	3	
	TC	24	1.88	0.99	2	−1	3	1	3	
	PLACEBO	23	1.7	1.11	2	0	3	1	2.5	
Flexion increase [cm]	LF	20	0.55	1.16	0	−0.5	3.5	0	1	*p* < 0.001 * η^2^ = 0.281 TC > HF, LF, Plac. HF > Plac.
	HF	24	0.67	0.94	0.5	−0.5	3	0	1	
	TC	24	1.46	1.02	1	0	5	1	2	
	PLACEBO	23	0.13	0.34	0	0	1	0	0	
Extension increase [cm]	LF	20	0.65	0.97	1	−1	2	0	1	*p* < 0.001 * η^2^ = 0.22 TC > LF, HF, Plac. LF > Plac.
	HF	24	0.33	1.32	0	−2	2	−0.62	1.62	
	TC	24	2.08	2.19	1.5	−2	6	1	3	
	PLACEBO	23	−0.04	0.93	0	−4	1	0	0	
Right lateral flexion increase [cm]	LF	20	0.65	0.8	0.25	0	2	0	1	*p* = 0.001 * η^2^ = 0.169 HF > LF, Plac. TC > Plac.
	HF	24	1.58	0.95	1.5	0.5	4	1	2	
	TC	24	1.29	1.27	1	0	4	0	2	
	PLACEBO	23	0.52	0.99	0	−1	3	0	1	
Left lateral flexion increase [cm]	LF	20	0.25	1.2	0.25	−1.5	3	−0.5	0.5	*p* = 0.009 * η^2^ = 0.098 TC, HF > LF, Plac.
	HF	24	1.33	1.39	1	0	5	0.38	2	
	TC	24	1.46	1.91	1	−1	5	0	3	
	PLACEBO	23	0.48	1.08	0	−1	3	0	1	
Right rotation increase [cm]	LF	20	1.05	1.04	1	0	3	0	2	*p* = 0.001 * η^2^ = 0.161 HF, TC, LF > Plac.
	HF	24	0.96	1.54	1.25	−2	3	0	2	
	TC	24	1.17	1.24	1	−2	3	0	2	
	PLACEBO	23	−0.13	0.76	0	−2	1	0	0	
Left rotation increase [cm]	LF	20	1.5	1.79	1	−1	6	1	2	*p* < 0.001 * η^2^ = 0.266 TC > HF, Plac. LF, HF > Plac.
	HF	24	0.92	1.47	1	−2	3	0	2	
	TC	24	1.96	1.57	2	−2	4	1	3	
	PLACEBO	23	0	0.52	0	−1	1	0	0	

*p*—Kruskal–Wallis test + post hoc analysis (Dunn test), SD—standard deviation, Q1—lower quartile, Q3—upper quartile; * statistically significant correlation (*p* < 0.05); η^2^—eta-squared.

**Table 7:** Comparison of positive long-term effects in specific groups.

Parameter	Group	N	Mean	SD	Median	Min	Max	Q1	Q3	*p*
VAS decrease	LF	20	3.75	1.12	3	2	6	3	4.25	*p* < 0.001 * η^2^ = 0.277 HF, LF > TC, Plac.
	HF	24	3.75	1.22	3.5	2	6	3	5	
	TC	24	2.58	1.41	3	−1	5	2	3	
	PLACEBO	23	1.52	1.59	2	−1	4	0	3	
Flexion increase [cm]	LF	20	0.7	1.34	0.5	−1	3.5	0	1.5	*p* = 0.004 * η^2^ = 0.12 HF > TC, Plac.
	HF	24	1.46	1.34	1	0	4.5	0.5	1.88	
	TC	24	0.21	2.04	1	−2	6	−2	1	
	PLACEBO	23	0.22	0.52	0	0	2	0	0	
Extension increase [cm]	LF	20	0.05	1.72	−0.5	−2	2	−1.5	2	*p* = 0.593 η^2^ = −0.013
	HF	24	−0.17	2.29	−0.25	−4	3	−2	2	
	TC	24	0.17	2.96	0.5	−5	5	−1	2	
	PLACEBO	23	−0.57	1.04	−1	−4	1	−1	0	
Right lateral flexion increase [cm]	LF	20	0.95	1.75	1.25	−3	3	0	2	*p* < 0.001 * η^2^ = 0.367 LF, HF, Plac. > TC
	HF	24	1.46	1.42	1	−1	4	0.88	2.25	
	TC	24	−1.08	1.28	−1	−3	1	−2.25	0	
	PLACEBO	23	1	0.95	1	0	3	0	1.5	
Left lateral flexion increase [cm]	LF	20	0.75	1.38	0.5	−1	3	−0.5	1.5	*p* < 0.001 * η^2^ = 0.229 HF > Plac., TC LF, Plac. > TC
	HF	24	1.58	1.43	1.25	−1	4	0.88	3	
	TC	24	−1.17	1.99	−2	−4	2	−2.25	1	
	PLACEBO	23	0.48	1.27	0	−1	3	0	1.5	
Right rotation increase [cm]	LF	20	1.15	1.26	1	−1	3	0	2	*p* < 0.001 * η^2^ = 0.246 LF > Plac., TC HF, Plac. > TC
	HF	24	0.54	1.47	1	−3	3	0	1	
	TC	24	−1.33	1.76	−1	−4	2	−3	0	
	PLACEBO	23	0	1.21	0	−2	2	−1	1	
Left rotation increase [cm]	LF	20	1.9	2.17	2.5	−2	6	0	3	*p* < 0.001 * η^2^ = 0.177 LF > TC, Plac. HF > Plac.
	HF	24	1	1.87	1	−4	3	0.75	2.25	
	TC	24	−0.04	2.14	0.5	−4	3	−1.25	1.25	
	PLACEBO	23	−0.26	1.1	0	−2	2	−1	0	

*p*—Kruskal-Wallis test + post-hoc analysis (Dunn test), SD—standard deviation, Q1—lower quartile, Q3—upper quartile; * statistically significant correlation (*p* < 0.05); η^2^—eta-squared.

**Table 8:** Analysis of intervention effects in comparison with the PLACEBO group using a Mixed-Effects regression model.

Parameter	Group	Est	z	*p*
VAS	LF	−0.19 ± 0.26	−0.76	*p* = 0.448
	HF	−0.35 ± 0.24	−1.45	*p* = 0.152
	TC	0.62 ± 0.24	2.53	*p* = 0.013 *
Flexion [cm]	LF	1.47 ± 0.23	6.43	*p* < 0.001 *
	HF	1.73 ± 0.22	7.92	*p* < 0.001 *
	TC	1.53 ± 0.22	7.03	*p* < 0.001 *
Extension [cm]	LF	0.71 ± 0.36	1.97	*p* = 0.052
	HF	0.86 ± 0.34	2.52	*p* = 0.014 *
	TC	0.02 ± 0.34	0.05	*p* = 0.962
Right lateral flexion [cm]	LF	−0.52 ± 0.3	−1.75	*p* = 0.084
	HF	−0.27 ± 0.28	−0.96	*p* = 0.34
	TC	−0.72 ± 0.28	−2.52	*p* = 0.013 *
Left lateral flexion [cm]	LF	−0.94 ± 0.32	−2.95	*p* = 0.004 *
	HF	−0.65 ± 0.3	−2.14	*p* = 0.035 *
	TC	−1.19 ± 0.3	−3.92	*p* < 0.001 *
Right rotation [cm]	LF	−0.07 ± 0.35	−0.19	*p* = 0.847
	HF	0.39 ± 0.34	1.15	*p* = 0.253
	TC	−0.25 ± 0.34	−0.74	*p* = 0.463
Left rotation [cm]	LF	−0.44 ± 0.33	−1.31	*p* = 0.193
	HF	0.27 ± 0.32	0.85	*p* = 0.398
	TC	−0.23 ± 0.32	−0.73	*p* = 0.469

Est—estimation parameter model, z—z-value; * statistical significance level (*p* < 0.05).

## Data Availability

Dataset available on request from the authors.

## Abbreviations

The following abbreviations are used in this manuscript:

NP	Neck pain
VAS	Visual Analog Scale
ROM	Range of motion
LF TENS	Low-frequency TENS
HF TENS	High-frequency TENS
TC	Träbert current
SSC	Science Club for Investigation of Physical Energy Used in Physiotherapy
NDI	Neck Disability Index
